# Proteome analysis of a recombinant *Bacillus megaterium *strain during heterologous production of a glucosyltransferase

**DOI:** 10.1186/1477-5956-3-4

**Published:** 2005-05-31

**Authors:** Wei Wang, Rajan Hollmann, Tobias Fürch, Manfred Nimtz, Marco Malten, Dieter Jahn, Wolf-Dieter Deckwer

**Affiliations:** 1 TU-BCE, German Research Centre for Biotechnology, Mascheroder Weg 1, D-38124 Braunschweig, Germany; 2 Department of Structural Biology, German Research Centre for Biotechnology, Mascheroder Weg 1, D-38124 Braunschweig, Germany; 3 Institute of Microbiology, Technical University Braunschweig, Spielmannstrasse 7, D-38106 Braunschweig, Germany

## Abstract

A recombinant *B. megaterium *strain was used for the heterologous production of a glucosyltransferase (dextransucrase). To better understand the physiological and metabolic responses of the host cell to cultivation and induction conditions, proteomic analysis was carried out by combined use of two-dimensional gel electrophoresis and mass spectrometry (2-DE/MS) for protein separation and identification.

2-DE method was optimized for the separation of intracellular proteins. Since the genome of *B. megaterium *is not yet available, peptide sequencing using peptide fragment information obtained from nanoelectrospray ionization quadrupole-time-of-flight tandem mass spectrometry (ESI-QqTOF MS/MS) was applied for protein identification. 167 protein spots were identified as 149 individual proteins, including most enzymes involved in the central carbon metabolic pathways and many enzymes related to amino acid synthesis and protein synthesis. Based on the results a 2-DE reference map and a corresponding protein database were constructed for further proteomic approaches on *B. megaterium*.

For the first time it became possible to perform comparative proteomic analysis on *B. megaterium *in a batch culture grown on glucose with xylose induction for dextrasucrase production. No significant differences were observed in the expression changes of enzymes of the glycolysis and TCA cycle, indicating that dextransucrase production, which amounted to only 2 % of the entire protein production, did not impose notable metabolic or energetic burdens on the central carbon metabolic pathway of the cells. However, a short-term up-regulation of aspartate aminotransferase, an enzyme closely related to dextransucrase production, in the induced culture demonstrated the feasibility to use 2-DE method for monitoring dextransucrase production. It was also observed that under the cultivation conditions used in this study *B. megaterium *tended to channel acetyl-CoA into pathways of polyhydroxybutyrate production. No expression increases were found with cytosolic chaperones such as GroEL and DnaK during dextransucrase production and secretion, whereas a strong up-regulation of the oligopeptide-binding protein OppA was observed in correlation with an increased secretion of dextransucrase into the culture medium.

## Background

The Gram-positive bacterium *B. megaterium *has been proven as a promising host for the production of diverse heterologous proteins and vitamins due to its intrinsic favourable properties such as low protease activity and high secretion capability [1]. Using recombinant *B. megaterium *strains for the heterologous production of a glucosyltransferase, namely dextransucrase from *Leuconostoc mesenteroides *NRRL B-512F, has been under investigation and improved production and secretion of dextransucrase was achieved compared with the recombinant production of dextransucrase in E. coli [2]. Dextransucrase can be used to catalyze polymerization reactions leading to the production of dextran. Dextran is widely used as a blood plasma substitute or as a basic chromatographic support material.

To optimize the cell cultivation and the recombinant protein production processes, it is important to understand the physiological and metabolic responses of the host cell to the cultivation and induction conditions. To this end we carried out proteomic analysis with a recombinant *B. megaterium *strain. Unlike *Bacillus subtilis*, which is the best characterized Gram-positive bacterium with its genome already completely sequenced in 1997 [3] and comprehensive proteomic analysis has been since accomplished [4-6], the genome of *B. megaterium *has not yet been sequenced and to our knowledge no information on proteomic analysis of *B. megaterium *has been published. In this work for the first time proteomic analysis of a recombinant *B. megaterium *strain based on two-dimensional gel electrophoresis in combination with mass spectrometric techniques (2-DE/MS) for protein separation and characterization was carried out. Comparative proteomic analysis was performed to study cellular protein expression changes related to defined cultivation and induction conditions for the production of recombinant dextransucrase by the recombinant *B. megaterium *strain.

## Results and discussion

### 1. Proteome mapping of the strain *B. megaterium *MS941*dsrS *by 2-DE/MS

**Figure 1:**
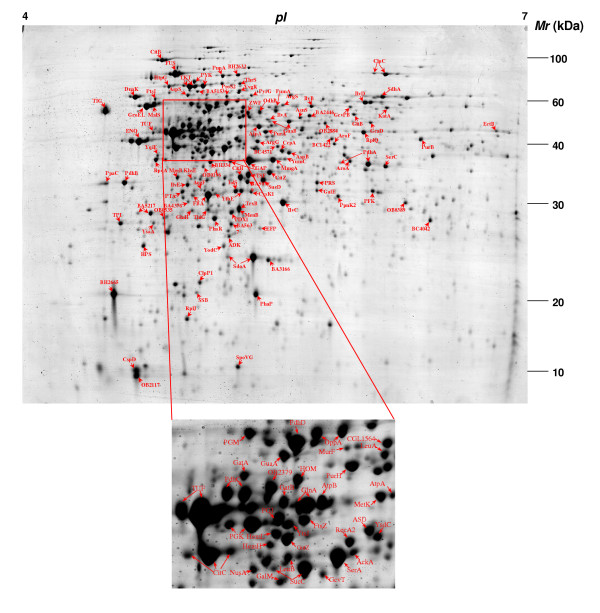
2-D IEF/SDS-PAGE gel electrophoretic separation of an intracellular protein extract of *B. megaterium *MS941*dsrS *at the pH range of 4–7. Cell sample was taken from a batch culture grown on glucose during the exponential growth phase and 6 h after xylose-induction. Proteins identified by ESI-QqTOF MS/MS are marked with their abbreviation names.

Methods used for the proteomic mapping of *B. megaterium *are the characterization of protein expression changes by 2-DE and the identification of proteins of interest by MS. This is aimed at establishing a functional metabolic network of *B. megaterium*, especially those involved in the central carbon metabolism, amino acid biosynthesis and protein biosynthesis, as well as the identification of metabolic pathways and cellular processes closely related to the production and secretion of the recombinant protein. Figure 1 shows a typical image of 2-DE separation of intracellular proteins of *B. megaterium *in the pH range of 4–7. When 250 μg of a protein sample were applied, about 580 – 800 protein spots can be detected on the different gels after coomasie staining, The spots matching rates between individual gels were between 58% to 75%.

#### 1.1 Peptide mass fingerprinting with MALDI-TOF MS

At present, the most straightforward and real high-throughput mass spectrometric method of protein identification is peptide mass fingerprinting. This approach of stringent matching of measured peptide masses with computer-generated masses relies, however, to a large degree on the availability of a completely sequenced genome of the investigated organism. Alternatively, if a protein of interest is not present in a database, peptide sequences deduced from tandem mass spectra can be employed for protein identification via sequence similarity searching. In this way a putative function can also be assigned to an unknown protein. Because of the rapid growth of completed genome sequences, it is becoming increasing possible to identify proteins of an unsequenced organism via sequence similarity searching, especially when sequences of organisms within the same kingdom or related species already exist in any of the public-accessible protein sequence databases [7-10].

In the case of *B. megaterium *its genome is not yet available. Up to now 76 protein sequences of *B. megaterium *can be found in the annotated protein database Swiss-Prot. However, only four of them are enzymes directly involved in the central carbon metabolic pathways, namely glyceraldehyde-3-phosphate dehydrogenase (GAP), 2,3-biphosphoglycerate-independent phosphoglycerate mutase (PGM), phosphoglycerate kinase (PGK) and triphosphate isomerase (TPI) for glycolysis. Another 215 protein sequences exist in the protein database TrEMBL, a computer-annotated supplement of Swiss-Prot . However, most of these proteins are hypothetical proteins and almost no enzymes of the central carbon metabolism or amino acid and protein biosynthesis are present. On the other hand, genome sequencing of several microorganisms from the genus *Bacillus*, i.e. *B. subtilis *[3], *B. halodurans *[11], *Oceanobacillus iheyensis *[12], *B. anthracis *[13], *B. cereus *[14] and recently *B. licheniformis *[15] have been finished. These *Bacillus *species show highly conserved orthologous genes, including those for central carbon metabolism and for amino acid and protein biosynthesis. In addition, as a model system of gram-positive bacteria the genome sequence of *B. subtilis *has been well annotated. Therefore, it is conceivable that the sequence information of these *Bacilli *can help the identification of unknown proteins of *B. megaterium *through sequence similarity searching.

At first, about 200 relatively highly expressed protein spots excised from 2-D gels were subjected to in-gel tryptic digestion and MALDI-TOF-MS analysis, followed by cross-species peptide mass fingerprinting against the protein sequence databases NCBInr and SWISS-PROT/TrEMBL. Regardless whether or not a constraint on species of origin was imposed, only 30 protein spots could be identified with significant scoring as 10 *B.megaterium *own proteins and 8 proteins showing high similarities to homologous proteins of other *Bacillus *species. Among the *B.megaterium *proteins identified are the four enzymes GAP, PGM, PGK and TPI present in the Swiss-Prot database.

#### 1.2 Peptide sequencing with ESI-QqTOF MS/MS

The result indicates that the homology between *B. megaterium *and other *Bacilli *species with completed genome sequences are still not high enough for an unambiguous identification of most of the unknown proteins of *B. megaterium *only through peptide mass fingerprinting. Consequently, ESI-QqTOF MS/MS analysis was carried out to acquire additional peptide sequence information for protein identification via sequence similarity searching for homologous proteins.

**Figure 2:**
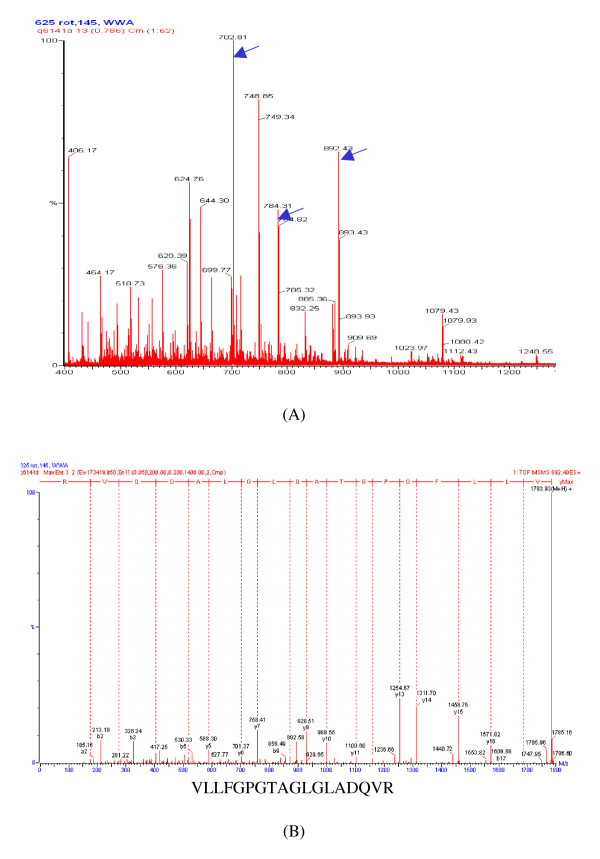
Sequencing of tryptic peptides by ESI-QqTOF MS/MS for protein identification via sequence-similarity searching. (A) MS spectrum of the peptide precursors of an unknown protein digested with trypsin, three double charged precursors of m/z 702.81, 784.31 and 892.43 were further fragmented to obtain their MS/MS sequence spectra; (B) MS/MS spectrum of the precursor m/z 892.43, shown at the bottom of the spectrum is the sequencing result of this peptide precursor.

Among the available sequence similarity searching programs using peptide sequences produced by MS/MS analysis, MS BLAST has been developed to overcome specific limitations imposed by mass spectrometric data, such as the limited completeness and confidence of predicted sequences. It is targeted at matching of closely related short peptides typically obtained from, for example, ESI-QqTOF MS/MS analysis [7-10,16]. However, the success of MS BLAST identification still depends on the number and quality of sequenced peptides. In our study, two to four tandem mass spectra of peptide precursors were normally available for a protein of interest from ESI-QqTOF MS/MS measurements. To obtain peptide sequences of better quality and to improve the possibility of protein identification, performing manual sequencing was still necessary for the interpretation of most of the MS/MS spectra. Peptide sequences obtained were assembled into query strings and subjected to MS BLAST searching. The results are presented as high-scoring pairs (HSPs) which are defined as regions of high local sequence similarity between individual peptides in the query and a protein sequence from the database entry. Homologous proteins shown in a hit list were sorted by their total scores, which are the sum of the scores of high-scoring pairs for each protein, and categorized according to their statistic significance into three groups: positive hit, borderline hit and negative hit. In this work we set the criteria for a positive identification as follows: the candidate protein is generally the top hit protein and the score of its top-ranked HSP should be higher than the statistic threshold. In addition, the candidate protein is normally a homologous protein from an organism of the genus *Bacillus*. Figure 2A shows, as an example, a MS spectrum of peptide precursors of an unknown protein digested with trypsin. Three double charged precursors of m/z 702.81, 784.31 and 892.43 (arrow indicated) were further fragmented to obtain their MS/MS spectra. Figure 2B is the MS/MS spectrum of the precursor m/z 892.43. Three amino acid sequences derived by manual sequencing of the three MS/MS spectra were used for similarity searching of homologous proteins. The NAD-dependent malic enzyme 3 of *B. subtilis *(MalS) was found as the top homologue protein candidate with a high score of 94 for the best aligned HSP and a total score of 242 which are significant enough for functional assignment of the unknown protein of *B. megaterium *as a malic enzyme.

About 300 relatively highly expressed protein spots were excised from 2-D gels and subjected to the ESI-QqTOF MS/MS analysis. 167 spot were identified as 149 individual proteins, because some proteins appeared as isoforms on the 2-D gels, namely as several spots having similar molecular weights (Mw) but different isoelectric points (pI). According to the categorization used in the KEGG PATHWAY database  most identified proteins can be classified into the following functional categories based on their functions or, at least, putative functions assigned: 53 proteins of the carbohydrate metabolism, mainly enzymes for the central carbon metabolism, including nearly all enzymes involved in the glycolysis and tricarboxylic acid cycle (TCA cycle), 4 enzymes of the pentose phosphate pathways, as well as 12 enzymes related to pyruvate metabolism; 31 proteins related to amino acid biosynthesis and metabolism; 14 proteins associated with protein biosynthesis; 15 proteins for nucleotide metabolism and genetic information (DNA, RNA) processing; as well as proteins involved in energy metabolism (3), cellular processes (5), membrane transport (9), stress responses (14) and other pathways like metabolism of complex carbohydrates (1), biosynthesis of secondary metabolites (2) and metabolism of cofactors and vitamins (3). Only 8 proteins can not be assigned any functions or putative functions. The identified proteins are summarized in Table 1 (see additional file 1).

**Figure 3:**
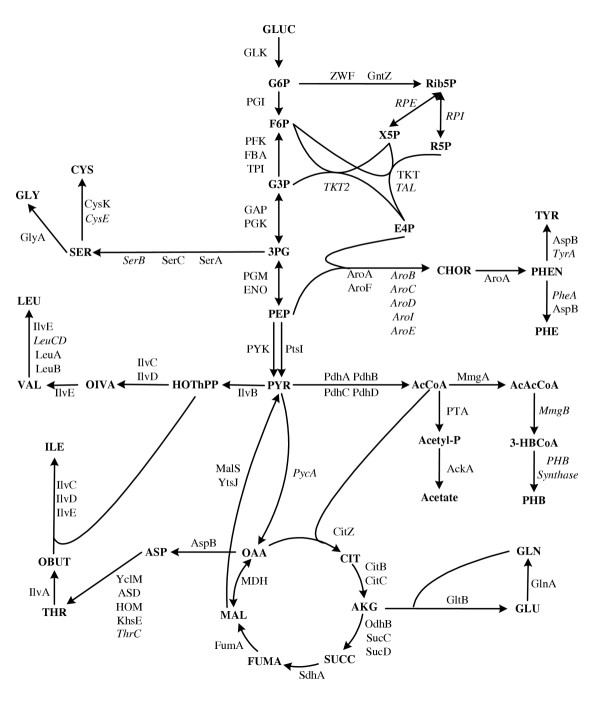
Metabolic Network of the central carbon metabolism and partial amino acid biosynthesis and metabolism of *B. megaterium *MS941*dsrS *constructed based on the identified proteins (italic labeled are proteins not yet identified).

This work represents the first effect of large scale 2-DE separation and quantification of intracellular proteins of *B. megaterium *combined with identification of many of these proteins by tandem mass spectrometric analysis. This enabled us to create a 2-DE reference map as highlighted in Figure 1 and a corresponding protein database of *B. megaterium*. Based on these information a metabolic network of the central carbon metabolism as well as a part of the amino acid biosynthesis and metabolism are constructed (Figure 3).

### 2. Batch culture of the *B. megaterim *strain MS941*dsrS *for recombinant dextransucrase production

**Figure 4:**
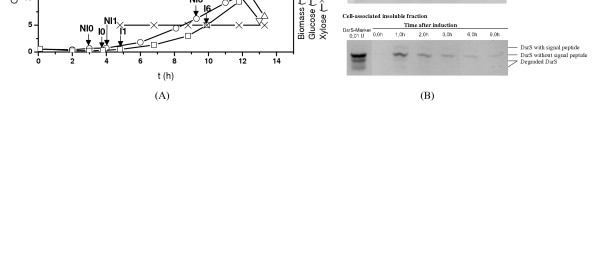
Batch cultures of *B. megaterium *MS941*dsrS *at pH 5.2. (A) Time course of cell growth on glucose. Induction was carried out with 5 g/L xylose at OD_578 _= 0.3 for the production of the recombinant dextransucrase (DsrS). Samples were taken for proteomic analysis shortly before induction (I0), then 1 h (I1), 6 h (I6) and 10 h (I10) after induction. Also shown is the time course of a control batch culture of *B. megaterium *MS941*dsrS *without xylose induction. Samples were also taken for proteomic analysis according to the sampling periods used for the induced culture. (B) Production, distribution and secretion of DsrS during the xylose induced batch culture. DsrS was detected as three cell-associated forms: precursor DsrS with signal peptide still attached, mature DsrS without signal peptide and degraded DsrS in three fractions: secretion, cell-associated soluble fraction and cell-associated insoluble fraction, respectively.

Our previous study on batch cultures of *B. megaterium *MS941*dsrS *revealed that an early xylose induction at OD_578 _of 0.3 and cultivation at pH 5.2 gave the best production and secretion of dextransucrase (data not published). Consequently, for proteomic analysis two batch cultures with B. *megaterium *MS941*dsrS*, one with xylose induction and the other one without xylose induction as control, were carried out under the same conditions. Conducting the control culture was aimed at distinguishing between protein expression changes resulting from recombinant dextransucrase production and those aroused from cell growth. As shown in Figure 4(A), the two batch cultures demonstrate very similar time course of cell growth and glucose consumption. Xylose added for induction was not consumed by the cells because of carbon catabolic repression by glucose.

To determine the formation and secretion of dextransucrase in *B. megaterium *cell samples were taken from the induced culture shortly before xylose induction, then 1 h, 2 h, 3 h, 6 h and 9 h after the induction. Three protein fractions, namely secretion fraction, cell-associated soluble fraction and cell-associated insoluble fraction, were prepared according to the method described in the Materials and Methods section and separated on SDS-PAGE activity staining gels to obtain dextransucrase in the different fractions. The results are shown in Figure 4(B). Three forms of dextransucrase of different molecular weights can be distinguished. They represent the precursor dextransucrase with signal peptide (200 kDa) that is still localized in the cytoplasm, the mature dextransucrase without signal peptide (188 KDa) that is already secreted outside the cell membrane and the degraded dextransucrase (165 kDa), respectively. A maximal production of dextransucrase reached already 1 h after the xylose induction which is shown as two thickest bands in the cell-associated soluble and insoluble fractions on the activity staining gels, respectively. In addition, the produced dextransucrase was readily translocated over the cell membrane, as is evidenced by the fact that no precursor dextransucrase is visible on the activity staining gels. However, dextransucrase aggregated in the space between cell membrane and cell wall, since no bands could be detected in the secretion fraction. The further passage of dextransucrase through the cell wall was hampered as it was also observed by Malten et al. [2]. Afterwards both cell-associated soluble and insoluble dextransucrase decreased steadily with time. Meanwhile the mature dextransucrase diffused continually through the cell wall to the surrounding growth medium. The diffusion barrier of the cell wall explains the time delay between maximal dextransucrase production and its maximal secretion into the medium which was reached 9 hours after induction.

### 3. Comparative proteomic analysis of metabolism

#### 3.1 Regulations of enzymes involved in the glycolysis and TCA cycle

**Figure 5:**
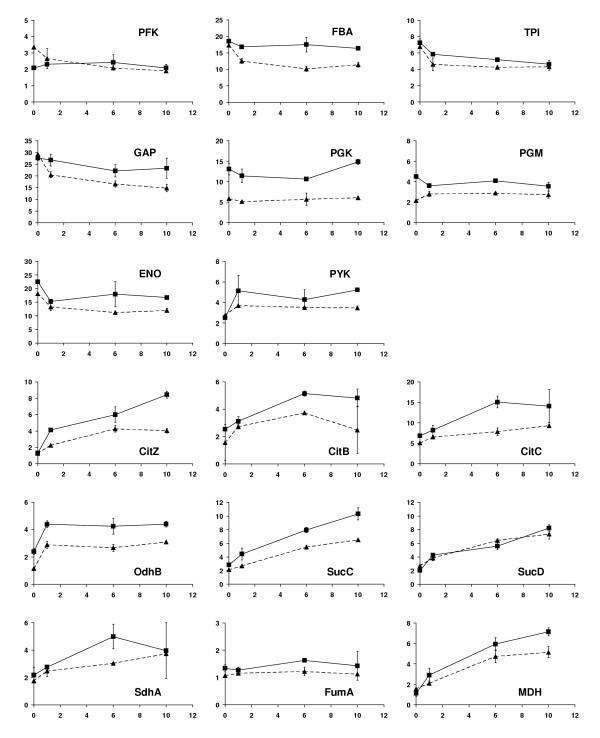
2-D electrophoresis determined expression changes of some enzymes involved in glycolysis (PFK, FBA, TPI, GAP, PGK, PGM, ENO and PYK) and TCA cycle (CitZ, CitB, CitC, OdhB, SucC, SucD, SdhA, FumA and MDH). The x-axis gives the time after xylose-induction and the y-axis gives the average value of normalized protein spot volume on 2-D gel. Abbreviations of protein names are referred to Table 1. Samples were taken from (A): a batch culture of *B. megaterium *MS941*dsrS *shortly before xylose-induction, then 1 h, 6 h and 10 h after induction for recombinant DsrS production (solid curves), and (B): a control batch culture of *B. megaterium *MS941*dsrS *without xylose-induction with the same time course of sampling as (A) (dashed curves).

As depicted in Figure 5 expressions of most enzymes of the glycolysis showed no obvious changes during the sampling period of cultivation. Slightly reduced expression levels can be observed 1 h after xylose induction, except for pyruvate kinase (PYK). The non-induced culture demonstrated a similar time course of expression. As also shown in Figure 5, except for fumarate hydratase (FumA) enzymes involved in the TCA cycle were up-regulated with most of them showing continuous increases up to 6 h after xylose induction. However, the batch culture without xylose induction showed again similar time courses of expression changes of the most TCA cycle enzymes. This indicates that the up-regulations were more likely a result of cell growth rather than a consequence attributed to the demand for the recombinant protein dextransucrase production. We found that during the first hour with maximal dextransucrase production only 6.75 mg dextransucrase / g CDW were built, compared to a biomass increase of 750 mg/L within this time span. Taken into account that the biomass of *B. megaterium *contains about 40% proteins [17], dextransucrase amounted to only 2% of entire proteins synthesized during this time period. Therefore, we believe that in this case the production of dextransucrase did not impose any noticeable metabolic and energetic burdens on the cells central carbon metabolic pathways.

**Figure 6:**
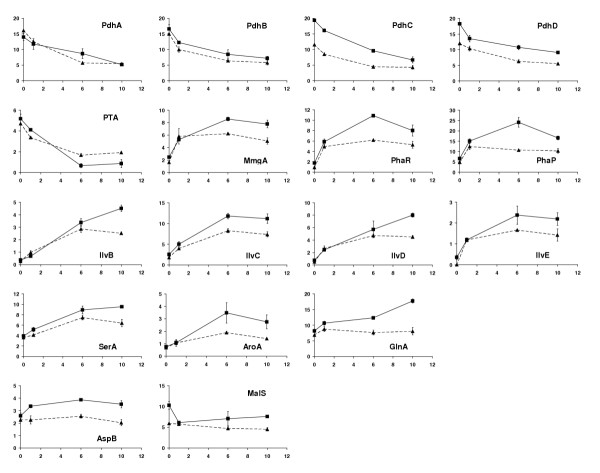
2-D electrophoresis determined expression changes of some enzymes involved in pyruvate metabolism (PdhA, PdhB, PdhC, PdhD and PTA), polyhydroxybutyrate synthesis (MmgA, PhaR and PhaP) and amino acid biosynthesis (AspB, GlnA, AroA, SerA, IlvB, IlvC, IlvD and IlvE), as well as the malic enzyme MalS. Additional descriptions for this figure are the same as given in Figure 5.

The expression of the enzyme pyruvate dehydrogenase was an exception. As shown in Figure 6, all subunits of the pyruvate dehydrogenase complex (PDH), the component E1 pyruvate dehydrogenase with its two subunits (PdhA and PdhB), the component E2 dihydrolipoamide acetyltransferase (PdhC) and the component E3 dihydrolipoamide dehydrogenase (PdhD) showed continuously decreased expression levels, especially in the early cell growth phase. As a key enzyme at the interface between glycolysis and TCA cycle, PDH is inhibited by ATP, NADH and its product acetyl-CoA. These findings propose that the reduced expression level of PDH was caused by an accumulation of acetyl-CoA in cells, indicating an inherent discrepancy between glycolysis and TCA cycle of this *B. megaterium *strain. Consequently acetyl-CoA generated could not be readily oxidized through the TCA cycle, even though many enzymes of TCA cycle were also up-regulated during this early growth phase.

#### 3.2 Regulations of enzymes involved in dissipation of acetyl-CoA

Contrary to the decreased expression of PDH, a notable up-regulation of acetyl-CoA acetyltransferase (MmgA), which converts acetyl-CoA to acetoacetyl-CoA, was observed in the early cell growth phase in both batch cultures (Figure 6), regardless whether or not there was a xylose induction. This can be considered as an additional evidence of the accumulation of acetyl-CoA in *B. megaterium *cells and how the cells cope with the oversupply of acetyl-CoA. It is well known that *B. megaterium *has a remarkable ability of producing polyhydroxyalkanoates (PHAs), a group of carbon and energy storage polymers that accumulate as inclusion bodies in many bacteria in response to environmental conditions [18-23]. Polyhydroxybutyrate (PHB) is the most common type of the family PHAs. *B. megaterium *cells taken from both induced and non-induced batch cultures were stained with Sudan black according to the method described by Burdon [24], and similar round PHB granule were visualized inside cells. Acetyl-CoA is used as substrate for the synthesis of PHB by a sequence of three reactions catalyzed by acetyl-CoA acetyltransferase (MmgA), 3-hydroxybutyryl-CoA dehydrogenase (MmgB) and poly(3-hydroxybutyrate) synthase (PHB Synthase). MmgA is the key regulatory enzyme of PHB synthesis from glucose [23]. Besides MmgA we have also identified two other proteins involved in PHB synthesis, namely PhaR and PhaP. PhaR functions directly or indirectly with another PHA inclusion body-associated protein PhaC to produce an active PHA synthase. It is required for PHA accumulation [20]. PhaP is characterized according to McCool [21] as a storage protein. It increased during the late lag phase and early to mid-exponential phase, decreased in mid- to late-exponential phase, and increased during stationary phase growth. PhaP belongs to the highly expressed intracellular proteins of *B. megaterium *as shown on the 2-D gels (Figure 1). In this work both PhaR and PhaP showed changes of expression levels similar to that of MmgA, namely a strong increase during the early cell growth phase both in induced and non-induced batch cultures (Figure 6). The expressions were then leveled off in the later growth phase in the batch culture without xylose induction, whereas in the induced batch culture the expressions increased further up to 6 h after xylose induction, then the expressions of PhaR and PhaP reduced from 6 h to 10 h after induction. Being a storage protein PhaP can be degraded as a source of amino acids for the cells [21]. Therefore, the decreased expression level of PhaP may imply its degradation for overcoming possible nutrient limitations and sustaining the cell growth in the stationary phase. The reason for the difference in expressions of MmgA, PhaR and PhaP between induced and non-induced cultures is not clear.

It is interesting that the phosphate acetyltransferase (PTA) which channels acetyl-CoA through acetyl phosphate into acetate under aerobic conditions was also strongly down-regulated in accordance with the decreased expression of PDH (Figure 6). This was verified by the very low concentration of acetate found in the culture medium. It seems that *B. megaterium *is in favor of using acetyl-CoA for PHB synthesis than switching on its overflow mechanism for the dissipation of the accumulated acetyl-CoA. This is receivable, since acetate accumulation is toxic for the cell, on the other hand accumulation of PHB is advantageous for the cell to store large quantities of carbon and energy without significantly affecting the osmotic pressure of the cell. In addition, PHB also serves as a sink of reducing power and could be regarded as a redox regulator within the cell. It has been even suggested that PHB may play a role in the regulation of intracellular calcium concentration and in calcium signalling in the plasma membrane of some Gram-positive and Gram-negative bacteria [23]. Indeed, the ability of *B. megaterium *to accumulate PHB is so dominant that the PHB content in the cells could reach up to 32% of the cell dry weight [19]. For the purpose of recombinant overproduction of dextransucrase it would be of interest to find out whether knock-out of PHB-producing genes could lead to better redirection of energy and carbon sources into the production of the heterologus protein in *B. megaterium*.

#### 3.3 Regulations of some enzymes involved in amino acid biosynthsis

As shown in Figure 6 four enzymes implicated in the synthesis of branched-chain amino acids valine, leucine and isoleucine using pyruvate as precursor were remarkably up-regulated. They are acetolactate synthase (IlvB), ketol-acid reductoisomerase (IlvC), dihydroxy-acid dehydratase (IlvD) and branched-chain amino acid aminotransferases (IlvE). Since the increases of the expression levels are comparable between the induced and the non-induced cultures, they should be resulted mainly from the demand on amino acids for cell growth. It is worth to mention that expression changes of these enzymes showed very similar time courses to the expression changes of MmgA, PhaR and PhaP as also shown in Figure 6. It has been reported [18] that branched-chain amino acids such as valine and isoleucine can induce the synthesis of phosphotransbutyrylase (YqiS), an enzyme which enhances the accumulation of PHB in *B. megaterium*. *B. megaterium *grown in culture media supplemented with valine and isoleucine showed drastically increased accumulation of PHB. Therefore, the enhanced expression levels of these enzymes appear to be related to the PHB synthesis and accumulation as discussed above.

Aspartate aminotransferase (AspB) is the enzyme that catalyzes the conversion of oxaloacetate to aspartate. Aspatate can be further converted to other amino acids of the oxaloacetate family. As shown in Figure 6, the expression of AspB remained at a quite constant level in the non-induced culture, whereas it showed about 50% increase 1 h after xylose induction in the induced culture. This was in agreement with the highest production of the recombinant dextransucrase during this time period as discussed above and can be due to the fact that aspartate, as well as threonine and asparagine of the oxaloacetate family belong to the most needed amino acids for dextransucrase synthesis. Amino acids of oxaloacetate family make together up to 37 % of the amino acid composition of dextransucrase. This demonstrates a high demand on the precursor oxaloacetate. Thus, the proteomic analysis can indicate the metabolic changes as shown for the short-term synthesis of dextransucrase.

The enhanced conversion of oxaloacetate to aspartate might be evidenced by the different expressions of the NAD-dependent malic enzyme MalS in the induced and non-induced cultures (Figure 6). While its expression maintained nearly constant in the non-induced culture, the expression of MalS decreased by 40% 1 h after xylose induction in the induced culture, indicating a reduced channeling of malate to pyruvate. During this time period the expression of malate dehadrogenase (MDH) increased by 160% in the induced culture compared with 36% increase in the non-induced cuture (Figure 5) which might also imply an enhanced conversion of malate to oxaloacetate for amino acid synthesis.

As was reported for *B. subtilis *[25,26] and *B. clausii *[27], malic enzymes form a "futile cycle" with pyruvate carboxylase (PycA) and malate dehydrogenase (MDH) (Figure 3). PycA fulfills an important role of catalyzing the anaplerotic reaction to directly replenish oxaloacetate from the pyruvate pool. Although PycA has not yet been identified from 2-DE/MS analysis in this work, our preliminary enzyme activity measurements using the method described by Schröder et al [28] has confirmed the existence of this enzyme in *B. megaterium *(data not published). It is conceivable that a similar "futile cycle" also exists in the metabolism of *B. megaterium*. Advantages of having this kind of "futile cycle" is still not clearly understood. It has been assumed that "futile cycle" is important for keeping the metabolic flexibility between the interface of glycolysis and TCA cycle. It plays a key role in anapleosis and metabolic regulations, especially for replenishing the TCA cycle under the stress of enhanced demand on precursors such as oxaloacetate and 2-oxoglutarate for biosynthesis. What an impact this kind of "futile cycle" may have on the production of the recombinant dextransucrase in *B. megaterium *will be further studied, not merely by proteomic analysis but in combination with metabolic flux analysis.

#### 3.4 Regulations of enzymes related to dextransucrase translocation

**Figure 7:**
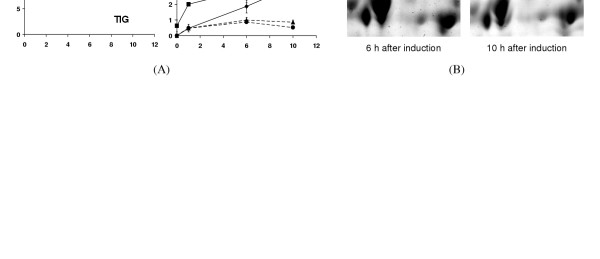
2-D electrophoresis determined expression changes of the chaperones GroEL, DnaK, TIG as well as the oligopeptide-binding protein OppA (A). Zoomed visualization of the expression alterations of the two OppA spots on the 2-D gels from samples taken from the xylose induced culture. Additional descriptions for this figure are the same as given in Figure 5.

It is often reported that microorganisms normally respond to the strong overproductions of recombinant proteins by increased expression levels of chaperones such as GroEL, DnaK and the trigger factor TIG [29-36]. However, from our proteomic analysis as shown in Figure 7 we found expression levels of these chaperones remained quite constant both in xylose-induced culture and in non-induced culture. This supports the observation that dextransucrase produced was readily translocated through the cytoplasmic membrane and therefore, required no over expression of these cytosolic chaperones. On the other hand, we observed an strong up-regulation of the oligopeptide-binding protein OppA in the induced culture compared with that in non-induced culture as also shown in Figure 7. Two isoforms of OppA were identified in this work. Expressions of both isoforms were steadily up-regulated after xylose induction. For a better visualization of the changes of the OppA spots zoomed sections on the 2-D gels are also shown in Figure 7. OppA is normally known as a component of the oligopeptide permease, a binding-protein dependent transport system. It plays an essential role in the uptake of peptides as nutrients and is also required for sporulation and competence for *B. subtilis*. The increased expression of OppA might be a kind of response of cells to the aggregation and eventual degradation of dextransucrase in the space between cell membrane and cell wall. In addition, competent cells are normally characterized by porous membranes that might facilitate further secretion of dextransucrase into the medium. This could be an explanation of the maximal secretion of dextransucrase happened 9 h after xylose induction as shown in Figure 4.

In addition, a chaperone-like property has been also suggested for the periplasmically located OppA of *E. coli *in addition to its function in transport by Richarme and Caldas [37]. They found that OppA interacts with unfolded and denaturated proteins to form stable complexes. It might bind a notable amount of unfolded proteins until permissive renaturation conditions are restored. Further the authors proposed that other binding proteins, including the substrate-binding lipoproteins of Gram-positive bacteria, should possess similar properties. Therefore, it would be of interest to further study the possible impact of OppA for the secretion of dextransucrase, so as to find out whether OppA is just an indicator of dextransucrase translocation and aggregation between the cytoplasmic membrane and the cell wall or it may act as an extracellular chaperone that can be exploited to help the passage of dextransucrase through the cell wall.

## Conclusion

In this work a 2DE method and a protein reference map for the proteomic analysis of *B. megaterium *were established. Despite the missing of genome sequence 149 individual proteins were identified through public protein database supported homologue protein searching using peptide fragmentation information acquired from ESI-QqTOF MS/MS analysis. Out of them 35 proteins could be assigned to enzyme functions of the central carbon metabolism (glycolysis, pentose phosphate pathway, TCA cycle and pyruvate metabolism) and 31 to amino acid synthesis and metabolism, leading to the construction of a partial metabolic network which is useful for metabolic pathway analysis.

During batch growth of *B. megaterium *on glucose expressions of glycolytic enzymes remained approximately constant, while most enzymes of the TCA cycle were up-regulated. The components of the PDH complex enzyme as well as phosphate acetyltransferase (PTA) were remarkably down-regulated, whereas some enzymes related to PHB synthesis were strongly up-regulated, indicating a metabolic discrepancy between glycolysis and TCA cycle of this *B. megaterium *strain and the channeling of acetyl-CoA into the biosynthesis of PHB as a carbon and energy storage source

Except for a few cases the protein expression profiles of the non-induced and induced *B. megaterium *batch cultures, the latter producing additionally the heterologous protein dextransucrase, did not differ significantly. This indicates that protein expression of *B. megaterium *concerning the central carbon metabolism was predominantly governed by growth and little affected by the xylose induced generation of the heterologous gene product. Indeed, the mass of dextransucrase was estimated as only 2% of the entire protein produced. Only some enzymes of amino acid synthesis exhibited discrepancies between induced and non-induced cultures. Specifically, the aspartate aminotransferase (AspB) was up-regulated in the induced culture. This enzyme channels oxaloacetate into a large family of amino acids which is strong required (37%) in dextransucrase synthesis. Expression levels of cytosolic chaperones needed for posttranslational processing hardly changed, whereas the oligopeptide-binding protein (OppA) exhibited increased expression in the induced culture, suggesting that this protein may be involved in the translocation of the heterologous dextransucrase.

## Materials and methods

### Batch cultivation of *B. megaterium *for recombinant dextransucrase production

The *B. megaterium *strain MS941 transformed with the plasmid pMM1520*dsrS *which carries the gene of a dextransucrase from *Leuconostoc mesenteroides *[38] was chosen as the candidate strain for the investigation on heterologous dextransucrase production. The medium used was developed for an optimal growth of the *B. megaterium *strain investigated in this study [39]. It contains per liter 33 g glucose monohydate, 5.0 g (NH_4_)_2_SO_4_, 2.2 g KH_2_PO_4_, 0.3 g MgSO_4_·7H_2_O, 0.5 g yeast extract, 2.0 mL trace element solution and 0.1 mL Sigma antifoam 204 per liter. The trace element solution contained 40 g MnCl_2_·4H_2_O, 53 g CaCl_2_·2H_2_O, 2.5 g FeSO_4_·7H_2_O, 2.5 g (NH_4_)_6_Mo_7_O_24_·4H_2_O and 2.5 g CoCl_2_· 6H_2_O.

At first, shaking flasks containing 50 mL of the medium were inoculated with a glycerol stock of the *B. megaterium *strain MS941 carrying the plasmid pMM1520*dsrS *(*B. megaterium *MS941*dsrS*) and cultivated overnight at 37°C and 250 rpm. Upon reaching an OD_578 _of approximately 3, 10 mL culture were added to 990 mL batch medium in a bioreactor (Biostat B2) and batch culture was carried out at 37°C, pH 5.2, 500 rpm and aeration of 1 L air/min. Analysis of biomass, glucose and metabolites were carried out as described in detail by Hollmann and Deckwer (2004). Biomass as cell dry weight (CDW) was calculated from measured OD value according to a linear relationship between OD and CDW that was determined in our previous works (data not published). Production of recombinant dextransucrase was induced by adding xylose at a concentration of 0.5% w/v after the OD_578 _reached about 0.3. For comparative 2-DE analysis, another batch culture without xylose induction was carried out under the same conditions.

For 2-DE analysis cell samples were taken from the xylose induced culture immediately before the induction (I0), then 1 h (I1), 6 h (I6) and 10 h (I10) after the induction. Similarly, after the OD_578 _reached about 0.3 the first cell sample (NI0) was taken from the non-induced culture. Further samples were then taken 1 h (NI1), 6 h (NI6) and 10 h (NI10) after the first sampling. Cells were immediately chilled in ice water after sampling, then centrifuged at 6500 rpm (Sorvall RT 6000B, DuPont) for 30 min at 4°C. Cell pellets were washed twice with phosphate-buffered saline (PBS) solution and stored at -80°C until use.

For the detection of the production and secretion of dextransucrase in *B. megaterium*, samples taken from the induced culture were centrifuged at 4°C and 5000 rpm for 10 min to obtain cell-free supernatants and cell pellets. The supernatants were centrifuged again at 4°C and 13000 rpm for 10 min to obtain protein sediments which contained the secreted dextransucrase as the secretion fractions. The cell pellets were resuspended with a lysis buffer (100 mM Na_3_PO_4_, 5 mg/mL lysozyme, pH 6,5 with H_3_PO_4_) and incubated at 37°C for 30 min and centrifuged at at 4°C and 13000 rpm for 10 min to separate the supernatant as the cell-associated soluble fractions from the sediments, which were then resuspended in 8M urea and centrifuged again to collect the supernatant as the cell-associated insoluble fractions. All three fractions were subjected to the determination of the activities of dextransucrase according to an activity staining method described by Malten et al [2]. Briefly, proteins of the three fraction were separated with normal SDS-PAGE gels. DsrS which was still bound to the gels was renaturated and incubated with sucrose to catalyze the formation of dextran. The amount of formed dextran was set into relation to the catalytic activity of DsrS for the quantification of the activity.

### Extraction and separation of intracellular protein of *B. megaterium *by 2-D IEF/SDS-PAGE

The 2-D IEF/SDS-PAGE gel electrophoresis (2-DE) method established in our laboratory for the proteomic analysis of different microorganisms [40] has been optimized for the separation of intracellular proteins of *B. megaterium*. To obtain raw protein extracts cell pellets were resuspended with a lysis buffer containing 7 M urea, 2 M thiourea, 4% (w/v) CHAPS, 1% (w/v) dithiothreitol (DTT), 0.8% (w/v) Pharmalyte™ pH 3–10, and 5 mM Pefabloc, and disrupted by ultrasonication in a ice bath for 5 × 60 s and 2 × 30 s with a 30 s interval between each ultrasonic cycle for better cooling effect. Insoluble materials were separated by centrifugation at 13,000 g for 30 min at 4°C. For an improved 2-DE separation of *B. megaterium *proteins, raw protein extracts should be further treated to diminish other interfering cell-intrinsic components. Different purification methods such as membrane dialysis using the Mini Dialysis Kits, 1 kDa cut-off (Amersham Biosciences), or precipitation with various organic solvents have been tested. A phenol precipitation with subsequent acetone extraction resulted in the best 2-DE separation performance. Briefly, raw protein extracts were extracted with a TE-buffer (10 mM EDTA, pH 7.4) saturated phenol by vigorous shaking and incubation. Proteins form a white interphase between the phenolic and the aqueous phases. After discarding the aqueous phase and washing the protein interphase twice with Milli-Q water, proteins were precipitated with cold acetone (-20°C), washed additionally two times with cold acetone, air-dried and stored at -80°C until use.

Aliquots of protein pellets were diluted with an adequate volume of rehydration buffer (7 M urea, 2 M thiourea, 4% (w/v) CHAPS, 1% (w/v) DTT, 0.5% IPG buffer pH 4–7 and trace amount of bromphenol blue). The total protein concentration in the supernatant was determined by the Bradford method using the RotiQuant reagent (Bio-Rad) according to the manufacturer's instruction. Duplicate gels were analyzed for each sample and all the samples from the same batch culture were run under the exactly same conditions. The first-dimensional gel electrophoresis of isoelectric focusing (IEF) was run with the IPGphor Isoelectric Focusing System (Amersham Biosciences) at a temperature of 20°C. 250 μg of each protein sample were loaded onto Immobiline DryStrip gels (IPG strips) of pH 4–7 by in-gel rehydration. IEF was performed with the following parameters: 30 V × 6 h, 60 V × 6 h, 200 V × 1 h, 500 V × 1 h, 1000 V × 1 h, gradient from 1000 V to 8000 V within 30 min, then 8000 V × 10 h. The second-dimensional gel electrophoresis of SDS-PAGE was carried out using the vertical slab separation unit Ettan Dalt II System and pre-cast polyacrylamide gels Ettan Dalt II Gel 12.5% (Amersham Biosciences). Prior to SDS-PAGE the IPG strips were equilibrated in the SDS equilibration buffer as recommended in the user's manual provided by the manufacturer for the pre-cast gels. SDS-PAGE separation was performed at 25°C in constant power mode as follows: 2 W/gel for 1 h and then 20 W/gel until the bromophenol blue dye front reached the bottom of the gel. Subsequently, gels were stained using Brilliant Blue G-Colloidal Concentrate (Sigma, Saint Louis, Missouri, USA) by fixing in a glacial acetic acid : methanol : water (7:40:53) solution for 1 h; staining overnight with Brilliant Blue G-Colloidal according to the manufacturer's instruction and rinsing several times with Milli-Q water. The gels were then scanned with a UMAX PowerLook III scanner at 300 dpi resolution to acquire the gel images. Computer analysis of the gels for protein spot detection, matching and quantification were performed with the Phoretix 2D Advanced Software Version 2003.02 (Phoretix, Newcastle upon Tyne, UK). Exactly same parameters such as sensitivity and operator size were used for spot detection. The same background subtraction method "lowest on boundary" was applied for all gels. User seeds were added to help spot matching between gels. To overcome the problem of electrophoretic variations between gels, minor manual adjustments during spot detection and matching were performed when it was obviously necessary. Average values of protein spot volume intensities and their corresponding standard deviations were calculated to characterize expression changes of proteins. Protein spot intensity was defined as the normalized spot volume which is the ratio of the single spot volume to the total spots volumes on a 2-D gel. Normally only proteins showing more than 2-fold increase or decrease in expressions were considered to be up- or down-regulated.

### Protein digestion and mass spectrometric analysis

Protein spots cut out from 2-DE gels were subjected to in-gel digestion with trypsin according to a method described previously [41] with some modification, namely, after rinse with water and dehydration with acetonitrile gel pieces were directly tryptic digested without further treatment. Peptides obtained were then extracted and purified with reversed-phased C_18 _ZipTips (Millipore, Bedford, USA).

Matrix-assisted laser desorption ionization time-of-flight mass spectrometry (MALDI-TOF MS) with a Bruker Ultraflex time-of-flight mass spectrometer (Bruker Daltonics GmbH, Germany) and nanoelectrospray ionization quadrupole-time-of-flight tandem mass spectrometry (ESI-QqTOF MS/MS) with a Q-TOF 2 mass spectrometer (Micromass, Manchester, England) equipped with a nanospray ion source were carried out as described by Wang et al [41].

### Protein identification by homologue protein searching

Peptide masses obtained from MALDI-TOF MS analysis were used for cross-species homologue protein searching in the public protein databases NCBInr and SWISS-PROT/TrEMBL by peptide mass fingerprinting. The search program Mascot (Matrix Science Ltd., UK, ) was used and search parameters were given as follows: trypsin was the digestion enzyme used, one missed cleavage sites was allowed, cysteine was modified by iodoacetamide and methionine was assumed to be partially oxidized. All peptide mass values are monoisotopic and the mass tolerance was set at 100 ppm.

MS/MS spectra of selected peptide precursors from ESI-QqTOF MS/MS analysis were enhanced using the Max Ent 3 software (Micromass), followed by automatic or manual sequencing using the PepSeq program of the software package Masslynx™ Version 3.5 (Micromass). Peptide sequences obtained were merged and submitted for similarity searching of homologous proteins using the protein database search program MS BLAST [7,16] with the scoring matrix PAM30MS and against the comprehensive non-redundant protein sequence database nrdb95. No constraints on protein molecular weights (Mw), isoelectric point (p*I*) or species of origin were imposed.

## Competing interests

The author(s) declare that they have no competing interests.

## Authors' contributions

W. Wang designed and carried out 2-DE experiments, did protein identification with MS data and contributed to the preparation of this manuscript. R. Hollmann and T. Fuerch carried out the batch culture experiments and the dextransucrase activity tests. M. Nimtz participated in ESI-QqTOF MS/MS and MALDI-TOF MS analysis as well as in discussions for the preparation of this manuscript. M. Malten and D. Jahn supplied the recombinant *B. megaterium *strain and were well involved in discussions for the preparation of this manuscript. W.-D. Deckwer initiated and coordinated this study, and contributed to the preparation of this manuscript. All authors have read and approved the final manuscript.

## Supplementary Material

Additional File 1 Table 1. Overview of intracellular proteins of *Bacillus megaterium *MS941*dsrS *separated by two dimensional gel electrophoresis and identified after in-gel tryptic digestion by ESI-QqTOF MS/MS analysis and homologue protein searching using MS BLAST against the non-redundant protein database nrdb95. Click here for file
