# Supplementary material for: Proteome analysis of a recombinant Bacillus megaterium strain during heterologous production of a glucosyltransferase
Source: Proteome Sci. 2005 May 31;3:4. doi: 10.1186/1477-5956-3-4 (PMC1175100; doi:10.1186/1477-5956-3-4)
Supplement: Additional File 1 — Table 1. Overview of intracellular proteins of Bacillus megaterium MS941dsrS separated by two dimensional gel electrophoresis and identified after in-gel tryptic digestion by ESI-QqTOF MS/MS analysis and homologue protein searching using MS BLAST against the non-redundant protein database nrdb95. [file 1477-5956-3-4-S1.pdf]

Table 1. Overview of intracellular proteins of *Bacillus megaterium* MS941*dsrS* separated by two dimensional gel electrophoresis and identified by in-gel tryptic digestion, ESI-QqTOF MS/MS analysis and homologue protein searching using MS BLAST against the non-redundant protein database nrdb95

Function category	Accession number	*pI* Db[a]/2-DE	*Mr*(KDa) Db/2-DE	Abbr. name[b]	Protein or homologous protein[c]	Peptide Sequence	High score	Total score
**Carbohydrate metabolism**								
Gycolysis / Gluconeogenesis	Q81K75	5.04/4.94	50.3/48.5	PGI	Phosphoglucose isomerase (Bacillus anthracis str. Ames)	DQAAGAEAFSGVFLEENPAYKYAVAR<br>TLVSEETTFTFVLPDLTGGR	90	118
	O34529	6.14/5.76	34.2/30.9	PFK	6-phosphofructokinase (Bacillus subtilis)	VAVLTSGGDSPGMNAPVR<br>LGVLTSGGDSPGFNAPVR	103	103
	P13243	5.19/4.90	30.4/30.0	FBA	Fructose-bisphosphate aldolase (Bacillus subtilis)	LNVNTENQLASAK	91	233
		5.19/4.97	30.4/29.9			LAAQPEQYDPR<br>EENSPVLLGVSEGAAR	91	233
	P35144	4.63/4.55	26.9/24.9	TPI	Triosephosphate isomerase (Bacillus megaterium)	EMFAETDETVNQK<br>SSTAEGANEVCAYLR<br>SVVAEQFSQDVADAVR	115	327
	X54520	5.18/5.18	35.9/39.0	GAP	Glyceraldehyde-3-phosphate dehydrogenase (Bacillus megaterium)	VLSWYDNESGYSNR<br>AVSLVLPELK	112	176
		5.18/5.07	35.9/38.9			VLSWYDNESGYSNR<br>LSLVLPELK<br>ENLLPTSTGAAK<br>LVSWYDNESGYSAAK	83	213
	P24269	4.95/4.85	42.4/47.7	PGK	Phosphoglycerate kinase (Bacillus megaterium)	AALPTLQYLVEQGAK<br>GVFELDAFADGTK	102	188
		4.95/4.89	42.4/47.7			AFAELADVYVNDAFGAAHR<br>ALSNPERPFTALVGGAK<br>VLLASHLGRPK	138	332

Table1. continued

Function category	Accession number	*pI* Db[a]/2-DE	*Mr*(KDa) Db/2-DE	Abbr. name[b]	Protein or homologous protein[c]	Peptide Sequence	High score	Total score
**Carbohydrate metabolism**								
Gycolysis / Gluconeogenesis	P35167	4.98/4.89	56.6/64.0	PGM	2,3-bisphosphoglycerate-independent phosphoglycerate mutase (Bacillus megaterium)	VNVALREGEFEQNETLLAAVK<br>ALQLSNTFANEDFR	150	257
	Q9K717	4.61/4.64	46.3/45.7	ENO	Enolase (2-phosphoglycerate dehydratase) (Bacillus halodurans)	ALVPSGASTGEYEAVELR	123	123
	P80885	5.06/4.91	62.2/79.3	PYK	Pyruvate kinase (Bacillus subtilis)	SPTGVELPAEEVPLVQK	98	98
		5.06/4.95	62.2/76.1			LVELLNSGLNVCGR<br>LGVELPAEEVPLVQK	103	133
	AE017271	6.07/4.95	39.3/40.2	GalM	Aldose 1-epimerase	PYFGAVVGR<br>LGGAAFELNNELYR	62	91
Pentose phosphate pathway	P45694	4.99/4.91	74.0/75.7	TKT	Transketolase (Bacillus subtilis)	LALESTDRPTALVLTR	92	92
		4.99/4.88	72.3/75.5		Transketolase (Bacillus cereus ATCC 14579)	GSEDFTAADYSGR<br>LVVLYDSQTLSLDATLDR	73	131
	P54547	5.41/5.16	55.7/58.8	ZWF	Glucose-6-phosphate 1-dehydrogenase (Bacillus subtilis)	LSEDFAVVGVAR<br>SLSVDEVDDYFVR<br>TESLDEQYNVPGNR	67	128
	P12013	5.25/4.96	51.8/45.6	GntZ	6-phosphogluconate dehydrogenase (Bacillus subtilis)	DYFGAHTYQR<br>NLALNLEDK<br>QQLGVLGLAVMGK<br>ALVDVLLDTAGQK	87	275
	Q9KGJ5	5.95/5.49	34.5/32.9	PRS	Ribose-phosphate pyrophosphokinase (Bacillus halodurans)	LNDDVVVVSPDHG	81	81

Table1. continued

Function category	Accession number	*pI* Db[a]/2-DE	*Mr*(KDa) Db/2-DE	Abbr. name[b]	Protein or homologous protein[c]	Peptide Sequence	High score	Total score
**Carbohydrate metabolism**								
Pyruvate metabolism	Q9K9J2	5.52 /5.60	41.4/38.4	PdhA	Pyruvate dehydrogenase (E1) alpha subunit (Bacillus halodurans)	FALSTPVEK<br>APALFVVQNNR<br>NGEGPTLLETLTYR	90	226
		5.05/5.74	40.1/37.9		Pyruvate dehydrogenase (E1) alpha subunit (Bacillus anthracis str. Ames)	APALFVVQNNR<br>AVDGEGPTLLETLTYR<br>QTFQLLNEEGEVVNK	95	274
	AF012285	4.74/4.87	35.4/32.4	PdhB	Pyruvate dehydrogenase (E1) beta subunit (Bacillus subtilis)	ATEGLQQEFGEDR<br>VVLPSTPYDAK<br>AQFTFLQALTDALR<br>TVQPLDLDTLLASVEK	94	338
	AF012285	5.04/4.85	47.5/52.6	PdhC	Dihydrolipoamide acetyltransferase (E2) (Bacillus subtilis)	LTFLPYVVK<br>LLNDPELLLMEA<br>SVFELSDAGLNDLAGK<br>FPALNTSLDDSTDEVLQK	108	290
		5.04/4.88	47.5/52.5			QDELDEDDVLAEVQNDK<br>ELDEDDVLAEVQNDQAVVELPSVPK	141	289
	P11959	5.20/4.98	49.3/61.7	PdhD	Dihydrolipoamide dehydrogenase (E3) (Geobacillus stearothermophilus)	ALALNAAEGFLK<br>VLDADYVLVTVGR<br>VGEETADGVKVTFEVGGESK	84	205
	Q81JR4	5.08/4.85	34.7/30.8	PTA	Phosphate acetyltransferase (Bacillus cereus ATCC 14579)	EEVVFVFPSLEAGNLGYK<br>TAEMFDLDPR	107	163
	P37877	5.34/5.08	43.1/43.7	AckA	Acetate kinase (Bacillus subtilis)	TALEVFASR	62	62
	O34389<br>P45868	5.08/4.71<br>5.07/4.71	62.1/63.9<br>64.1/63.9	MalS<br>YwkA	Probable NAD-dependent malic enzyme 3 (Bacillus subtilis)<br>Probable NAD-dependent malic enzyme 2 (Bacillus subtilis)	LAVYTAAAGLDPSR<br>AYEQFLAQADNLGK<br>VLLFGPGTAGLGLADQVR	94<br>94	242<br>239

Table1. continued

Function category	Accession number	*pI* Db[a]/2-DE	*Mr*(KDa) Db/2-DE	Abbr. name[b]	Protein or homologous protein[c]	Peptide Sequence	High score	Total score
**Carbohydrate metabolism**								
Pyruvate metabolism	O34962	5.23/4.96	43.7/47.7	YtsJ	Probable NAD-dependent malic enzyme 4 (Bacillus subtilis)	SFAGVDAFPLALDTM<br>TTFPNQVNNVLAFPGLF	111	201
	Q8CX93	7.69/5.28	41.5/39.3	MmgA	Acetyl-CoA acetyltransferase (Oceanobacillus iheyensis)	TEVVLVSAVR<br>LVLFGNVLQAGLGQNAPR	88	149
	Q9ZF94	5.04/5.00	22.9/25.6	PhaR	PhaR (Bacillus megaterium)	TQGEQLQAQLLEQEAGK	90	90
	Q9ZF96	5.31/5.20	19.9/17.3	PhaP	PhaP (Bacillus megaterium)	ALEQQQEFVTK<br>TRQEFQHVSDAYVEQVK	131	212
TCA cycle	Q9K847	5.49/5.26	41.5/35.6	CitZ	Citrate synthase II (Bacillus halodurans)	LQAQLPTVVTAFSR<br>DPEADLFDEEANYR<br>PYSDLQTVENAEPYLR	82	196
	P09339	5.09/4.75	99.3/98.0	CitB	Aconitase (Bacillus subtilis)	ALTAPVSNQSFGLDAK<br>FVEFFGPGVAELPLADR<br>QDFTGVPAVVDLASLR	118	229
	P39126	5.26/4.85	46.9/43.9	CitC	Isocitrate dehydrogenase [NADP] (Bacillus subtilis)	YFDGVPSPLK<br>NETEDLYAGLEYAK<br>LETGEWLPEETLDALR	86	226
		5.03/4.82	46.4/43.7			VVTYDFAR<br>AALNYALEQGR<br>LVSFLQTELGVNK	65	190
		5.03/4.78	46.4/43.4			LVSFLQTELGVNK<br>DQTWWLPEETLDALR	65	129
	P16263	5.05/5.22	46.0/59.6	OdhB	Dihydrolipoamide succinyltransferase (Bacillus subtilis)	TFAGLEGSLVDLAK<br>CFNEVDFTAVFALR<br>KLSPLGLAVSAVLGLVVPVVR	90	151

Table1. continued

Function category	Accession number	*pI* Db[a]/2-DE	*Mr*(KDa) Db/2-DE	Abbr. name[b]	Protein or homologous protein[c]	Peptide Sequence	High score	Total score
**Carbohydrate metabolism**								
TCA cycle	Q81WK0	5.00/4.93	41.7/41.7	SucC	Succinyl-CoA synthetase beta chain (Bacillus anthracis str. Ames)	LVGLSLPLVVR	64	64
		5.04/4.99	41.7/41.7		Succinyl-CoA synthetase beta chain (Bacillus halodurans)	LNFDSNALYR<br>VAFTVEEAVEAAK<br>ATSQVVLFGSEEGGTELEEVAEQTPEK	158	323
	Q9KA21	5.34/5.24	31.3/33.6	SucD	Succinyl-CoA synthetase Alpha subunit (Bacillus halodurans)	SVFLNQDTK<br>VLQEQGLYEQCGK<br>GFLGGQTAPPGK	82	163
	Q81L77	5.69/5.81	65.9/68.5	SdhA	Succinate dehydrogenase, flavoprotein subunit (Bacillus anthracis str. Ames)	WSNQAATFTR<br>RAGASLLSALYGFGVAGPK	63	122
	Q04718	5.26/5.30	56.8/62.0	FumA	Fumarate hydratase class I (Geobacillus stearothermophilus)	GFGGETTLLG	65	65
	Q81KZ7	5.12/5.13	33.5/34.2	MDH	Malate dehydrogenase (Bacillus anthracis str. Ames)	VLGQSGVLDSAR<br>YSYAGGLPLETLLPK<br>TFVAQELNLSVK	102	252
Other carbohydrate metabolism	Q81QH9	5.85/5.46	27.1/57.4	BA2446	N-acetylmuramoyl-L-alanine amidase (Bacillus anthracis)	TEDLFVLSNSK<br>AFLDRDDLFVLSNSK	54	108
	P46336	5.50/5.10	35.2/30.9	IolS	IOLS protein (Bacillus subtilis)	SLGVSNFSLDQLK<br>LVGDELLLDNSPAFLK<br>ALDDGLNLLDTAFLYGPK	80	188
	Q81XS7	4.97/4.92	64.1/72.0	BA5153	Phosphomannomutase (Bacillus anthracis str. Ames)	SLLNQGVVLAYDSR<br>SPNPEEPAAFEYAVR	97	155
	Q8EMG3	4.98/5.53	49.7/53.7	OB2884	UDP-glucose 6-dehydrogenase (Oceanobacillus iheyensis HTE831)	YASNDFLALK	70	70

Table1. continued

Function category	Accession number	*pI* Db[a]/2-DE	*Mr*(KDa) Db/2-DE	Abbr. name[b]	Protein or homologous protein[c]	Peptide Sequence	High score	Total score
**Carbohydrate metabolism**								
Other carbohydrate metabolism	Q9K7L7	5.06/4.98	42.4/38.6	BH3344	NADH-dependent butanol dehydrogenase (Bacillus halodurans)	VLLLYGGGSVK<br>YDGDVWDLLNR<br>MCGEGVLQTVLETGPK	81	211
	Q81KC2	5.28/5.16	36.9/33.2	BA5079	Oxidoreductase (Bacillus anthracis str. Ames)	VSDELEDTSSR<br>LLNDYSADATLR	82	137
	P55180	4.98/5.49	37.0/31.1	GalE	UDP-glucose 4-epimerase (Bacillus subtilis)	TATYNLGDGLGYSVK	54	54
	Q9KGN6	4.98/5.08	31.9/26.8	PDX1	Pyridoxine biosynthesis protein pdx1 (Bacillus halodurans)	TKGEPGTGNLVEAVRNVLM<br>DVLNAEQAK	110	191
	AAR39392	5.24/4.66	22.6/21.8	HPS	3-hexulose-6-phosphate synthase (Bacillus subtilis)	AQPDLVLVGGGLTSQDDQK<br>FELQLALDLVNLSQAK	84	164
**Amino acid biosynthesis and metabolism**								
	P26901	6.15/5.83	54.6/61.8	KatA	Catalane (Bacillus subtilis)	SVYYEPNSFGGPTESKENK	114	114
	O32214	4.76/5.12	67.2/72.5	YvgR	Sulfite reductase [NADPH] flavoprotein alpha-component-like protein (Bacillus subtilis)	VVNSPFNQEQADLLNR<br>LQPGDTLPVYLQSNK<br>DLDFEEPANK	103	232
	AX100793	5.44/5.72	59.5/65.0	IlvD	Dihydroxy-acid dehydratase (Bacillus subtilis)	QVAVLFGNLAPVGSLLK<br>LNLPTVFVSGGQ	75	144
	AX100787	5.32/5.47	62.6/62.6	IlvB	acetolactate synthase large subunit (Oceanobacillus iheyensis)	QPVLLAGAGVLLSK<br>QEGQELFYEER	64	125
	P54377	5.40/ 5.65	54.4/62.2	GcvPB	Probable glycine dehydrogenase [decarboxylating] subunit 2 (Bacillus subtilis)			

Table1. continued

Function category	Accession number	*pI* Db[a]/2-DE	*Mr*(KDa) Db/2-DE	Abbr. name[b]	Protein or homologous protein[c]	Peptide Sequence	High score	Total score
**Amino acid biosynthesis and metabolism**								
	Q9K9S5	5.15/5.07	49.2/61.0	MurF	UDP-N-acetylmuramoylalanyl-D-glutamyl-2,6-diaminopimelate-D-alanyl-D-alanyl ligase (Bacillus subtilis)	TLQQGNLYLPLQGER<br>TFLNDAYNASPTSVK	84	153
	Q9K8E8	5.50/5.14	56.2/59.6	LeuA	2-isopropylmalate synthase (Bacillus stearothermophilus)	LLGDLLEAGFPASSK<br>LQSLNGGPDALAEVFVK	73	146
	O34399	7.94/5.67	54.9/58.1	GltB	Glutamate synthase (Bacillus subtilis)	VLGSGPAGLASADQLNQAGH<br>NNFPEFTGR<br>NTVEGVFAAGDAR	121	258
	Q9KC63	5.64/5.26	46.5/55.4	IlvA	Threonine deaminase (Bacillus halodurans)	VVLLGDTFDDSYEQA	68	68
	Q814M4	4,99/4.98	46.8/55.0	HOM	Homoserine dehydrogenase (Bacillus cereus ATCC 14579)	FFGLVDGTTNFLLTK<br>VQGLTEVTEEDLQYSK	81	161
	Q81KI0	5.20/5.13	43.2/52.0	MetK	S-adenosylmethionine synthetase (Bacillus anthracis str. Ames)	QTAAYGHFGR<br>FVLGGPQGDAGLTGR	104	179
	M22811	5.05/5.00	50.3/50.7	GlnA	Glutamine synthetase (Bacillus subtilis)	NVELPVSQLEK<br>SVDPAANPYLAFAVLLK<br>LVPGYEAPHEGGLADSAR	92	215
		5,04/4.98	50.1/50.0		Glutamine synthetase, type I (Bacillus anthracis str. Ames)	LVPGYEAPGCYVAWSAR	58	116
	P94417	4,97/4.98	49.8/50.0	YclM	Probable aspartokinase (Bacillus subtilis)	NANYENFTDVDALYSVDPR	104	104

Table1. continued

Function category	Accession number	*pI* Db[a]/2-DE	*Mr*(KDa) Db/2-DE	Abbr. name[b]	Protein or homologous protein[c]	Peptide Sequence	High score	Total score
**Amino acid biosynthesis and metabolism**								
	Q9KA89	5.19/ 5.11	38.0/46.9	ASD	Aspartate-semialdehyde dehydrogenase (Bacillus halodurans)	KAWNSVQLAESLLK<br>ETYQAVSGAQAALNEL	74	130
	Q81FZ0	5.53/5.56	42.7/46.8	BC1422	Sulfate adenylyltransferase (Bacillus cereus ATCC 14579)	QLVDGLFLNPLVGETK	102	102
	Q8GCA5	4.96/4.94	46.4/46.2	HemL	Glutamate-1-semialdehyde 2,1-aminotransferase (Bacillus megaterium)	SQRATLGLPDSPGVPEGLAK<br>VTESGTSFGATPLLENELAK	112	224
	Q81KV7	5.20/ 5.21	44.7/45.8	ArgG	Argininosuccinate synthase (Bacillus anthracis str. Ames)	VVLAYSGGLDTWALK<br>NLLPPLNLDSPFSLDQNLWGR	124	197
	Q81SV7	5.51/ 5.58	42.5/44.8	AroF	Chorismate synthase (Bacillus subtilis)	TVEELQEVTEASPVR	68	68
	P32396	4.81/4.93	35.3/44.7	HemH	Ferrochelatase (Bacillus subtilis)	LNEVQDELEFK	62	62
	P05644	5.36/4.96	39.6/43.1	LeuB	3-isopropylmalate dehydrogenase (Bacillus caldotenax)	VAVLPGDGVGTEVTK<br>ELTGGLYFGQP	78	153
	Q81N95	5.93/5.05	43.1/43.1	SerA	D-3-phosphoglycerate dehydrogenase (Bacillus anthracis str. Ames)	YLTDFPNQNVLNMK<br>FNQEGYQLDNDSENPDALVVR	88	88
	P53001	5.43/5.36	43.1/41.7	AspB	Aspartate aminotransferase (Bacillus subtilis)	LSYATSLDLLTR<br>DQGLTYSPSELLV	63	113
	Q81M06	5.15/5.02	40.2/41.0	GcvT	Aminomethyltransferase (Bacillus cereus ATCC 14579)	FEATLALYGQELSK	88	88

Table1. continued

Function category	Accession number	*pI* Db[a]/2-DE	*Mr*(KDa) Db/2-DE	Abbr. name[b]	Protein or homologous protein[c]	Peptide Sequence	High score	Total score
**Amino acid biosynthesis and metabolism**								
	P39912	5.48/5.65	40.2/38.9	AroA/ PheB	Phospho-2-dehydro-3-deoxyheptonate aldolase/chorismate mutase (Bacillus subtilis)	TSPYDFQGLGVEGLK NFQNFELLK	99	153
	Q9KDM4	6.01/5.82	39.5/37.3	SerC	Phosphoserine aminotransferase (Bacillus halodurans)	EQMYTPHATEESR FYQSKFSLLYAGAQK	58	138
	Q81RS0	5.24/ 4.89	32.1/33.9	KhsE	Homoserine kinase (Bacillus anthracis str. Ames)	YGVSLSGAGPSL QVPASSANLG	61	108
	AX100818	5.13/ 4.87	40.3/32.4	IlvE	Putative branched-chain amino acid aminotransferase (Bacillus subtilis)	GYSQVLWLDGVER	76	76
	P18186	5.13/ 4.94	34.7/31.2	ArgF	Ornithine carbamoyltransferase (Bacillus subtilis)	LFAPYQVNEELAPHAK	75	75
	Q81VX2	5.37/5.18	32.9/30.7	CysK-1	Cysteine synthase A (Bacillus anthracis)	LEFFNPGSSVK ALLTFPETFTVER	66	117
	Q9K8E7	5.22/5.33	37.1/28.5	IlvC	Ketol-acid reductoisomerase (Bacillus halodurans)	ETLVEAGYQPEVAYFVEVL SWDQAEQDGFTVLSVK YSLSDTAQVSGDFVSGPR	113	267
	Q819E3	6.41/6.04	32.0/25.4	BC4042	3-hydroxyisobutyrate dehydrogenase (Bacillus cereus ATCC 14579)	CPETGAAGSWSLSNLPVK	80	80
**Protein biosynthesis**								
Ribosome	Q81VU1	5.38/4.87	18.0/15.8	RplJ	Ribosomal protein L10 (Bacillus anthracis str. Ames)	LQASNSLVVVDYR	57	57

Table1. continued

Function category	Accession number	*pI* Db[a]/2-DE	*Mr*(KDa) Db/2-DE	Abbr. name[b]	Protein or homologous protein[c]	Peptide Sequence	High score	Total score
**Protein biosynthesis**								
Translation factors	Q9F4B2	5.13/4.81	77.2/85.6	FUS	Translation elongation factor G (Geobacillus stearothermophilus)	LQEEDPTFR	69	69
	Q9Z9L6	4.80/4.80	43.4/49.0	TUF	Elongation factor Tu (Bacillus halodurans)	KLLDYAEAGDNLGALLR	115	115
	Q81VT2	4.93/4.77	42.9/49.6		Elongation factor Tu (Bacillus cereus ATCC 14579)	FTAEVYVLSK<br>VGDVLDLLGLTEEPK	73	127
	P33166	4.92/4.71	43.6/49.3		Elongation factor Tu (Bacillus subtilis)	EVLSEYDFPGDDVPVLK	107	107
	Q81WK9	5.25/5.16	32.4/34.9	TSF	Elongation factor Ts (Bacillus anthracis str. Ames)	QADAFGAYLHQGR<br>ALTKTDGDFEKALYVLR	67	126
	P49778	5.03/5.22	20.4/24.1	EFP	Elongation factor P (Bacillus subtilis)	TGLTVEVDGGLWR	82	82
Aminoacryl-tRNA biosynthesis	P18255	5.37/5.12	74.1/77.8	ThrS	Threonyl-tRNA synthetase (Bacillus cereus ATCC 14579)	LSLYQQGEFFDLCGR	90	90
	O32038	4.85/4.87	66.0/69.4	AspS	Aspartyl-tRNA synthetase (acillus subtilis subsp. subtilis str. 168)	TPPFALADQTEVSED<br>AYDLVLDGYE	67	198
	Q81WL6	5.00/5.06	63.2/68.6	ProS-2	Prolyl-tRNA synthetase (Bacillus anthracis str. Ames)	QNSSGVYSFLPLGK<br>VGSVGPLGVSDSVEVVADD<br>FLQEGDQSPDGQGVLK	85	228
	Q81JT7	5.27/5.33	62.5/63.4	ArgS1	Arginyl-tRNA synthetase 1 (Bacillus anthracis str. Ames)	LAEELVANFDK	77	77

Table1. continued

Function category	Accession number	*pI* Db[a]/2-DE	*Mr*(KDa) Db/2-DE	Abbr. name[b]	Protein or homologous protein[c]	Peptide Sequence	High score	Total score
**Protein biosynthesis**								
Other carbohydrate metabolism	Q9KC78	5.38/5.41	49.1/56.3	AsnS	Asparaginyl-tRNA synthetase (Bacillus halodurans)	VFSFGPTFR<br>DGSGFFQGVVVK<br>SFTQESSLYVLASVR	69	196
	Q81ZE8	5.16/4.88	52.3/55.6	GatA	Glutamyl-tRNA amidotransferase subunit A (Bacillus anthracis str. Ames)	LLANFDPLYDATVVQK<br>YALATYYLLSSSEASANLSR	135	249
	O30509	5.09/4.95	53.6/51.0	GatB	Aspartyl/glutamyl-tRNA amidotransferase subunit B (Bacillus subtilis)	VLLSGGLLEQETR<br>PCLMVALTPEGLAGFLQLLQEGTLSSK	106	160
Peptidases	Q817H8	5.37/5.30	39.7/43.9	BC4571	Deblocking aminopeptidase (Bacillus cereus ATCC 14579)	YLAPYADEVTTDGLGSLLAK	92	92
	P54542	4.95/4.73	39.6/40.8	YqjE	Hypothetical protein yqjE (Bacillus subtilis)	LDEETTANLGR	76	76
**Nucleotide metabolism and genetic information processing**								
	Q8ELX5	5.35/5.33	85.1/85.6	OB3089	Ribonucleoside-diphosphate reductase alpha subunit (Oceanobacillus iheyensis HTE831)	YPDNVLELNDLPVLQAK	60	60
	U2966	5.08/5.04	77.5/83.8	PnpA	Polynucleotide phosphorylase (Bacillus subtilis)	ELLDEALQQAK<br>FVVNPTVDELEK	72	140
	Q9K6D7	5.34/5.18	59.2/69.3	PyrG	CTP synthase (Bacillus halodurans)	CPGLQPNVLVVR	69	69
	P29727	4.94/4.95	58.0/59.4	GuaA	GMP synthase (Bacillus subtilis)	LPWDVLEVLSTR	87	87

Table1. continued

Function category	Accession number	*pI* Db[a]/2-DE	*Mr*(KDa) Db/2-DE	Abbr. name[b]	Protein or homologous protein[c]	Peptide Sequence	High score	Total score
**Nucleotide metabolism and genetic information processing**								
	Q81ZG8	5.35/5.07	55.5/57.5	PurH	Phosphoribosylaminoimidazolecarboxamide formyltransferase/IMP cyclohydrolase (Bacillus anthracis str. Ames)	ELSYNNLNDANAALQLVK	105	105
	Q9RHG1	6.34/5.36	55.2/54.5	ImpDH	Inosine-5'-monophosphate dehydrogenase (Bacillus cereus)	LTAVYDCGATEAR<br>EGLTFDDVLLVPAK<br>EAYPELNLLAGNVATAE<br>PAGVGVPQLTAVYD	96	366
	X55669	6.31 /5.27	55.7/54.4	GuaB	IMP dehydrogenase (Bacillus subtilis)	SGVLTDPFFLTN<br>EAYPELNLLAGNVATVTG	96	161
	P29726	5.67/5.25	47.9/49.7	PurA	Adenylosuccinate synthetase (Bacillus subtilis)	RVGWFDSVVVR<br>VSQLTGLPLSLFSVGDPR	96	182
	P12047	5.88/5.99	49.5/46.6	PurB	Adenylosuccinate lyase (Bacillus subtilis)	NPLGSENVTLGAR<br>LGLSPAPLSTQTLQR	78	133
	P20429	4.80/4.72	34.8/39.4	RpoA	DNA-directed RNA polymerase alpha chain (Bacillus subtilis)	GYGTTLGNSLRR	82	82
	P38372	4.74/5.07	24.2/23.0	ADK	Adenylate kinase (Bacillus halodurans)	FNLVLFGLPGAGK<br>QSQPLLDFYQEK<br>NLTANQDLNLVFEDVR	71	190
	Q8GJ70	5.08/5.07	37.4/45.1	RecA2	RecA2 protein (Bacillus megaterium)	VGVFFGNPETTPGGR<br>VVEVYGPESSGK	97	183
	P46828	5.31/5.33	36.6/42.3	CcpA	Glucose-resistance amylase regulator (Bacillus megaterium)	MNVTLYDVAR<br>GLNVPNDLELLGFDNTR<br>NLAFVSGTLEEPLPPGAK<br>VDGNPNVQH	125	339

Table1. continued

Function category	Accession number	*pI* Db[a]/2-DE	*Mr*(KDa) Db/2-DE	Abbr. name[b]	Protein or homologous protein[c]	Peptide Sequence	High score	Total score
**Nucleotide metabolism and genetic information processing**								
	P32727	4.79/4.90	41.7/41.7	NusA	Transcription elongation protein nusA (Bacillus subtilis subsp. subtilis str. 168)	GPELYDGTVELK	79	79
	P37455	5.02/4.92	18.7/17.4	SSB	Single-strand binding protein (Bacillus subtilis)	YTPSGAAVATFTLAVNR	113	113
**Energy metabolism**								
	P17674	5.33/5.15	54.6/52.4	AtpA	ATP synthase alpha chain (Bacillus megaterium)	VVNSLGQPVDGLGPVETTK	131	131
		5.33/5.22	54.6/52.1			ALDALVPLGR<br>LMEVPVGEQLLGR<br>EAYPGDVFYLHSR	100	268
	P12698	5.05/5.02	51.3/51.0	AtpB	ATP synthase beta chain (Bacillus megaterium)	VFNVLGEQLDLDAPLDAGAR<br>FENLSTQAELLETGLK<br>EVEDTGAALSVPVGDVTLGR	136	370
	P37487	4.73/4.48	34.0/32.7	PpaC	Manganese-dependent inorganic pyrophosphatase (Bacillus subtilis)	LGDLNGETQFALEK<br>LANFETADPLYYR<br>LEAELAGVDAEEYGLAFLQ	92	273
**Other metabolisms**								
Biosynthesis of sencondary metabolites	O34678	4.96/5.13	31.8/31.0	YtbE	Putative morphine dehydrogenase (Bacillus subtilis)	VWNADLGYEETLAA	103	103
		4.96/5.00	31.8/30.3			DTAALYGNEEGVGEGLR<br>ADLGYEETLAAYETSLA	108	211
	Q65IF9	5.39/5.04	22.6/21.8	YodC	YodC (putative nitroreductase) (Bacillus licheniformis)	LLPLAYNQK<br>YLPVFLLSVGK	60	114

Table1. continued

Function category	Accession number	*pI* Db[a]/2-DE	*Mr*(KDa) Db/2-DE	Abbr. name[b]	Protein or homologous protein[c]	Peptide Sequence	High score	Total score
**Other metabolisms**								
Metabolism of complex carbohydrates	P28017	5.82/5.75	49.3/54.0	GcaD	UDP-N-acetylglucosamine pyrophosphorylase (Bacillus megaterium)	YAVLLAAGQGTR	80	80
Metabolism of cofactors and vitamins	O34934	6.45/5.59	30.2/29.7	PpnK2	Probable inorganic polyphosphate/ATP-NAD kinase 2 (Bacillus subtilis)	LGLDNEALNAR	58	58
	P23966	5.47/5.12	30.0/27.3	MenB	Naphthoate synthase (Bacillus subtilis)	VGSFDAGYGSGYLAR<br>YEDLLYETYDGLAK	105	190
	O31618	4.91/4.97	27.0/27.1	ThiG	Thiazole biosynthesis protein thiG (Bacillus subtilis subsp. subtilis str. 168)	VLPYTSDDVLLAR	85	85
**Cellular processes**								
	P17865	5.01/4.99	40.4/48.4	FtsZ	Cell division protein ftsZ (Bacillus subtilis)	GLGAGANPEVGKK<br>VLGVGGGGNNAVNR	97	183
	Q8ETR1	5.57/4.95	38.5/37.2	OB0196	ATP-binding Mrp-like protein (Oceanobacillus iheyensis HTE831)	SFGFFVEDNSPVLWR	99	99
	Q01465	5.09/4.82	35.9/34.2	MreB	Rod shape-determining protein mreB (Bacillus subtilis)	GLVLTGGGALLR<br>TPPELAAD<br>LAGDEMDEALLQYLR	85	216
	P28016	5.28/5.11	10.7/13.8	SpoVG	Stage V sporulation protein G (Bacillus megaterium)	GQLQDAVLAEYHR<br>ALASLTLDGEFVVHDLR	124	220
	Q81LE2	4.82/4.89	29.1/27.0	MinD	Septum site-determining protein MinD (Bacillus anthracis str. Ames)	LLGLLEQEDLESPK	73	73

Table1. continued

Function category	Accession number	*pI* Db[a]/2-DE	*Mr*(KDa) Db/2-DE	Abbr. name[b]	Protein or homologous protein[c]	Peptide Sequence	High score	Total score
**Membrane Transport**								
	O69251	4.77/4.72	63.6/67.0	PtsI	Phosphoenolpyruvate-protein phosphotransferase (Bacillus megaterium)	CMELPAVVGTK<br>SVTEVEAEVAR<br>MEFPFLATVDEFR	78	221
	P24141	5.61/5.06	61.5/65.0	OppA	Oligopeptide-binding protein oppA (Bacillus subtilis)	NESQYAYQLYYVK<br>VQLDNSEQEVYLEK	98	157
		5.61/5.01	61.5/64.3			CGQGSLDDVAVK<br>VQLDNSEWAVYLEK	83	146
	Q8NQ75	4.85/5.14	53.5/62.0	CGL1564	Iron-regulated ABC-type transporter SufB (Corynebacterium glutamicum ATCC 13032)	VDTPLQAYFR<br>VSEEQLFYLMSR<br>LNSAVWSG HVAPY	91	247
	Q5WJ28	4.05/4.94	39.1/34.5	ABC1089	Sugar ABC transporter substrate-binding protein (Bacillus clausii)	NPDAEVLSQYAESF	79	79
	Q81XL7	4.67/4.63	29.0/26.5	BA5217	ABC transporter, ATP-binding protein (Bacillus anthracis str. Ames)	YLNEGFSGGEK<br>STLSSALMGHPK<br>FLAFQYPSELSGVTN<br>TQGSLTLDDADVLEFEVDER	136	194
		4.67/4.69	29.0/26.5			QLFLAFQYPSELSGVTNADFLR<br>YLNEGFSGWK	95	353
	Q9KCV8	4.01/4.87	30.0/28.7	BH1461	ABC transporter, Substrate-binding protein (Bacillus halodurans)	NVDFSDVYYEAK<br>TVGVQTSSLQADEAK	73	126
		4.01/4.89	30.0/27.0			VDFSDVYYEAK	73	73
	Q81M76	6.53/4.87	28.7/28.7	BA4376	Amino acid ABC transporter, amino acid-binding protein (Bacillus anthracis str. Ames)	DRLPELLQEFK<br>VLVFGTSADY	63	121

Table1. continued

Function category	Accession number	*pI* Db[a]/2-DE	*Mr*(KDa) Db/2-DE	Abbr. name[b]	Protein or homologous protein[c]	Peptide Sequence	High score	Total score
**Membrane Transport**								
	O34563	8.30/4.89	29.8/27.0	GlnH	Glutamine ABC transporter, Glutamine-binding protein (Bacillus subtilis subsp. subtilis str. 168)	VDFSDVYYEA	71	71
**Stress response**								
	Q9KGG2	5.86/5.83	90.6/85.1	ClpC	Class III stress response-related ATPase (Bacillus halodurans)	VLALAQEEALR<br>VFTLDFGTVVAGTK<br>AESLFGDEDAFLR	86	230
	P46208	4.86/4.78	72.2/75.6	HtpG	Chaperone protein htpG (Bacillus subtilis)	GFVDSEDLSLNLSR<br>LLEFFLNSLYSQK<br>ELLSNSSDALDQLYYK	100	256
	Y00154	4.69/4.61	65.2/68.3	DnaK	Chaperone protein dnaK (Heat shock protein 70) (Bacillus megaterium)	FQLTDLPPAPR<br>AKFDELSAGLVER	89	172
	Q81VE1	4.79/4.68	57.4/62.5	GroEL	60 kDa chaperonin (Protein Cpn60) (Bacillus anthracis str. Ames)	VGNDGVLTLEESK<br>FGSPLLTNDGVTLAK<br>TTVVDGAGNAEDLLAR<br>GLVAGGGTALVNLYNK	100	321
	Q8CXB7	4.42/4.48	48.2/48.9	TIG	Trigger factor (Oceanobacillus iheyensis HTE831)	EQGNKGVLTVEVLSAEFNK	62	62
	P5086	4.84/4.77	46.4/49.6	ClpX	ATP-dependent Clp protease ATP-binding subunit clpX (Bacillus subtilis subsp. subtilis str. 168)	TLLAQTLAR	57	57
	Q9K9M1	6.71/6.35	49.7/50.1	BH2624	Aminotransferase (diaminobutyrate--pyruvate transaminase) (Bacillus halodurans)	NLPLLVDEVQTGLGR<br>LLPPLLLTK	96	139

Table1. continued

Function category	Accession number	*pI* Db[a]/2-DE	*Mr*(KDa) Db/2-DE	Abbr. name[b]	Protein or homologous protein[c]	Peptide Sequence	High score	Total score
**Stress response**								
	Q65FE0	5.35/5.34	36.8/39.5	YumC	YumC (FAD-dependent pyridine nucleotide-disulphide oxidoreductase) (Bacillus licheniformis DSM 13)	YLYDVAGFPK<br>LLVAGFGEGPTAVNNAK	99	180
	Q9K703	5.25/5.12	34.3/27.3	TrxB	Thioredoxin reductase (NADPH) (Bacillus halodurans)	DVENYPYGDHLLDGPLSNK<br>RSYCGAVCGDGAFFK	55	100
	Q9ZF38	5.21/5.06	22.5/20.6	SodA	Manganese superoxide dismutase (Bacillus licheniformis)	FGSFDQFQEEFANAAK<br>SNLDAVPEDLR<br>NEELLSNLDAVPEDLR	86	171
	Q9KD10	5.29/5.19	22.4/20.5		Superoxide dismutase [Mn](Bacillus halodurans)	RPDYLSAFFNVVK<br>PLLGLDVWEHAYYLNNL<br>SLEELLSNLDAVPEDLR	113	278
	Q8VQM7	5.26/4.93	21.4/18.2	ClpP1	ATP-dependent Clp protease proteolytic subunit (Bacillus thuringiensis)	TGQPLEVLQR<br>RDDTDNFFTAER	99	99
	Q9K9I2	4.55/4.52	20.8/17.3	BH2665	2-cys peroxiredoxin (Bacillus halodurans)	EYGVLLEEEGVALR<br>GGDLQYPLAADTNHVVSR<br>YLLSPEWLQYSVVNHNNLGR	190	405
	Q81K90	4.60/4.63	7.2/13.8	CspD	Cold shock-like protein cspD (Bacillus anthracis)	TLEEGQEVTFELVEGNR	116	116
	Q8EPI6	4.37/4.63	11.6/13.6	OB2117	Thioredoxin (Oceanobacillus iheyensis)	FGVFSLPTLVLFK	70	70

Table1. continued

Function category	Accession number	*pI* Db[a]/2-DE	*Mr*(KDa) Db/2-DE	Abbr. name[b]	Protein or homologous protein[c]	Peptide Sequence	High score	Total score
**Unknown**								
	Q9K9L3	4.96/5.11	69.0/84.6	BH2632	GTP-binding protein TypA/BipA (Bacillus halodurans)	VGQQVALFK<br>EGYELQVSKLAAPNAR	65	137
	Q8ENU7	4.96/4.93	48.8/53.6	OB2379	Hypothetical conserved protein (Oceanobacillus iheyensis HTE831)	QPTAAYSTLTEELQSQGVLFTMPQTAAK	71	71
	Q65GB8	5.73/5.12	39.3/46.0	YsdC	Peptidase M42 family YsdC (Bacillus licheniformis)	YLAPYADEVTTDGLGSLLAK	105	105
	Q8ET73	5.53/5.92	32.5/28.5	OB0389	2,5-diketo-D-gluconate reductase (Oceanobacillus iheyensis HTE831)	ALPTLQESLYR<br>NLQLESWSPLGR<br>GNEGVNALTSALDVGYR	72	183
	Q8EST2	4.77/4.76	31.6/25.7	OB0535	Oxidoreductase (Oceanobacillus iheyensis HTE831)	EQLFLTTK<br>VWNSDQGYENALR	73	129
	Q81JR3	4.99/5.08	28.6/24.7	BA5637	Hypothetical protein (Bacillus anthracis str. Ames)	EAEQQGSHALYTVVS<br>KGNDNWYFLPFEER	76	134
	P94512	4.86/4.71	29.5/24.9	YsaA	Hypothetical protein ysaa (Bacillus subtilis)	VLHDLDDTLLWDQK	75	75
	Q81NM2	4.89/5.26	23.4/20.2	BA3166	Isochorismatase family protein (Bacillus anthracis str. Ames)	EVFVVTDASGTFNK<br>DYGVDQFQNNVLALGDTAK	99	184

[a] **Db** indicates isoelctric point (*pI*) and molecular weight (*Mr)* calculated from the amino acid sequence of the identified B. megaterium´s own protein or of a homologue protein, whereas **2-DE** indicates *pI* and *Mr* of the protein derived from 2-DE experiments.
[b] **Abbr. name** indicates abbreviation of a protein name derived from its gene name used in the KEGG PATHWAY database. For an unknown gene name it is the ordered locus name of the corresponding homologue protein.
[c] **Protein** refers to B. megaterium´s own protein and **Homologue Protein** are homologue proteins of other *Bacillus* species.
